# Numerical Simulation and Field Monitoring of Blasting Vibration for Tunnel In-Situ Expansion by a Non-Cut Blast Scheme

**DOI:** 10.3390/s24144546

**Published:** 2024-07-13

**Authors:** Zhenchang Guan, Lifu Xie, Dong Chen, Jingkang Shi

**Affiliations:** College of Civil Engineering, Fuzhou University, Fuzhou 350108, China; zcguan@fzu.edu.cn (Z.G.); 220527193@fzu.edu.cn (L.X.); 220520109@fzu.edu.cn (D.C.)

**Keywords:** in-situ tunnel expansion, non-cut blast scheme, numerical simulation, field monitoring, vibration effect

## Abstract

There have been ever more in-situ tunnel extension projects due to the growing demand for transportation. The traditional blast scheme requires a large quantity of explosive and the vibration effect is hard to control. In order to reduce explosive consumption and the vibration effect, an optimized non-cut blast scheme was proposed and applied to the in-situ expansion of the Gushan Tunnel. Refined numerical simulation was adopted to compare the traditional and optimized blast schemes. The vibration attenuation within the interlaid rock mass and the vibration effect on the adjacent tunnel were studied and compared. The simulation results were validated by the field monitoring of the vibration effect on the adjacent tunnel. Both the simulation and the monitoring results showed that the vibration velocity on the adjacent tunnel’s back side was much smaller than its counterpart on the blast side, i.e., the presence of cavity reduced the blasting vibration effect significantly. The optimized non-cut blast scheme, which effectively utilized the existing free surface, could reduce the explosive consumption and vibration effect significantly, and might be preferred for in-situ tunnel expansion projects.

## 1. Introduction

**Figure 1:**
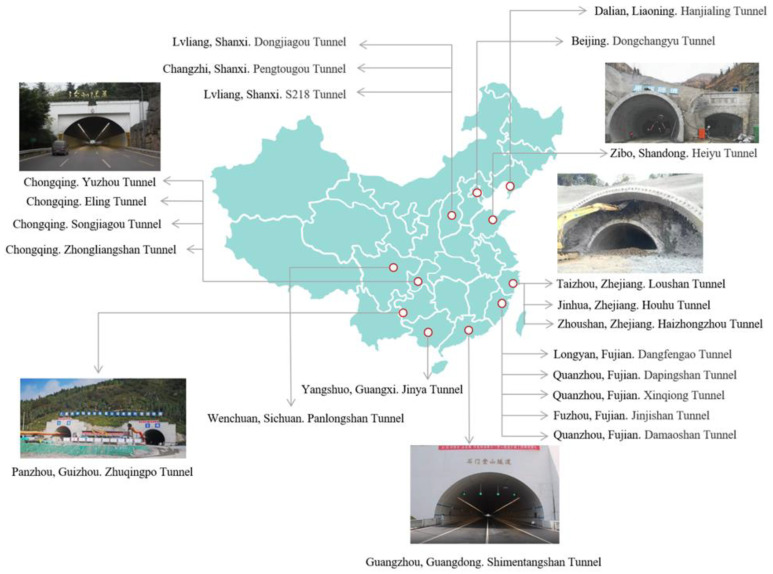
The engineering practices of tunnel reconstruction or expansion.

Traffic volume has increased rapidly in past decades with rapid economic development in China, and a large number of existing infrastructures have become insufficient to cope with the growing traffic volume. As far as tunnels are concerned, reconstruction or in-situ expansion projects have become more and more popular in recent years (Figure 1) to meet the growing demand for traffic capacity. Similar tunnel expansion projects can also be seen outside China, such as the Lowari Tunnel in Pakistan [1], the Castellano tunnel in Italy [2] and the M2 Motorway tunnel in Sydney, Australia [3].

The drilling and blasting method still plays an important role in tunnel reconstruction or expansion projects due to its flexibility and cost-efficiency. However, blasting can lead to a great number of negative effects, including the cracking of concrete lining [4], the loosening of surrounding ground [5,6,7,8,9,10], and negative effects on adjacent structures, such as existing tunnels [11,12], pipelines [13,14,15,16], airports [17], and ground buildings [18,19,20]; the impact of tunnel blasting on adjacent existing tunnels is the focus of this paper.

The primary indicators utilized to assess the impact of tunnel blasting on adjacent tunnels are the response mechanism of the lining itself and the vibration variation law of surrounding rock mass. For the lining’s response mechanism, Dang et al. [21] and Zhao et al. [22] studied the impact of new tunnel blasting on the safety of existing tunnels’ lining using PPV and vibration frequency as safety evaluation indexes in combination with numerical simulation and field monitoring, and the research results can provide theoretical guidance for similar projects. Through a series of numerical simulations on LS-DYNA, Han et al. [23] and Zhang [24] obtained the lining damage modes under different blast-load levels and the variation characteristics of the maximum principal stresses and PPV of adjacent tunnel linings during the excavation of existing tunnels, respectively. Wang et al. [25] used Sadovsky’s empirical formula to determine the PPV attenuation law of old and new tunnel linings based on field monitoring data from an in-situ expansion project. Ma et al. [26] realized vibration signal extraction and analysis of tunnel linings and other structures in in-situ expansion projects by developing a CEEMD-MPE-HT time-frequency analysis model. Zhou et al. [27] conducted a study on the damage mechanism of tunnel lining structures under blasting loads during the excavation of an adjacent metro station using LS-DYNA. The study revealed that the lining structures experienced tensile damage when the blasting tensile stresses significantly exceeded their tensile strength. Utilizing the tunnel expansion project as an example, Zhang et al. [24] leveraged LS-DYNA to analyze the dynamic response of the adjacent tunnel lining during the blasting and excavation of an existing tunnel, using the maximum principal stresses and PPV as criteria. By combining field monitoring and numerical simulation for railway cross tunnels, Zhao et al. [28] presented a method for evaluating the blasting vibration response of existing tunnels with a full section. This method can serve as a guide for the blasting vibration control of similar projects. Utilizing hard rock tunnels as an engineering background, Zhou et al. [29] investigated the vibration effect of adjacent structures (tunnels, slopes, etc.) during tunnel blasting construction. Additionally, the blasting plan was refined by optimizing the blasting parameters, and field tests confirmed that the refined plan made sense.

For the vibration variation law of surrounding rock mass, Dang et al. [21] concluded from 3D numerical simulation that the damage to surrounding rock mass was primarily concentrated around the new tunnel. An et al. [30] used a numerical simulation method that combined finite and discrete elements to study the rupture of surrounding rock and debris accumulation caused by blasting, and proved the superiority of the method through actual engineering monitoring data. Song et al. [31] designed a large-scale dimensional tunnel excavation simulation test system to explore the dynamic response of the interlayer surrounding rock during the excavation of a large cross-section tunnel with small clearance. The results indicated that the blasting process during the backward tunnel excavation had a greater influence on the stability of the interlaid rock. Qiu et al. [32] derived the strain distribution on the model surface and the failure pattern of the surrounding rock under different blasting positions by conducting a series of model tests. Wang et al. [33] predicted the PPV on adjacent tunnel section considering the diffraction and reflection amplification effects of blast-induced vibration around the adjacent tunnel.

The two evaluation indicators above are closely related to the blast scheme design in underground excavation. Sharafat et al. [11] proposed an innovative approach to limit the range of explosion damage by incorporating critical particle velocity into the vibration attenuation model. Kang and Jang [34] evaluated the blast damage zone by considering both the rock mass condition and the blasting mechanism. Jia et al. [35] established three models to predict blast vibration velocity and enhance the explosion quantity by considering the free surface effect. Minh et al. [36] optimized the contour blasting parameters by numerical simulations and optimization algorithms. Iwano et al. [37] optimized the blast scheme by using advanced electronic detonators to reduce the vibration effect on the adjacent environment.

The traditional blast scheme in previous studies, however, required a large number of explosives, and it was difficult to control the vibration effects. Therefore, this paper proposed a non-cut blast scheme, aiming to reduce the explosives used in the blast and the vibration effect on the surrounding environments. The rest of this paper was structured as follows: first, the Gushan Tunnel was introduced as the engineering background for the whole; second, the traditional blast scheme and the non-cut blast scheme were analyzed and compared through refined numerical simulations; third, the non-cut blast scheme was selected and applied to engineering practice; fourth, field monitoring was carried out to assess the vibration effect on the adjacent environment and validate the efficiency of the non-cut blast scheme for an in-situ tunnel expansion project; finally, several conclusions were drawn.

## 2. Expansion of the Gushan Tunnel

### 2.1. General Situation

The Gushan Tunnel is located in the east district of Fuzhou city and serves as a significant part of the second ring road from 1987. It consists of two separate tunnels with lengths of 968 m and 936 m, respectively, and accommodates four lanes in two directions. Due to early construction and long-term heavy-duty operation, some problems appeared in the Gushan Tunnel, including insufficient thickness of the arch lining, incomplete arch back, and water seepage into the lining. At the same time, rapid economic development also led to the actual traffic volume far exceeding the design capacity of the tunnel after three decades. Therefore, the in-situ expansion project was carried out from March 2018 to June 2020 to accommodate eight lanes in two directions.

**Figure 2:**
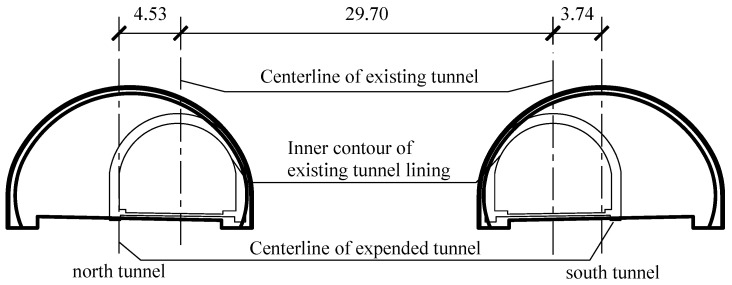
The typical cross-section of the Gushan tunnel before and after in-situ expansion (unit: m).

The typical cross-section of the Gushan tunnel before and after expansion (taking mileage K18+110 as an example) is shown in Figure 2. The expansion of the north tunnel was carried out first (from March 2018 to May 2019) and bidirectional transportation was temporarily shifted to the existing south tunnel with two lanes. It was succeeded by the expansion of the south tunnel (from May 2019 to June 2020) and bidirectional transportation was temporarily shifted to the expanded north tunnel with four lanes. The cross-section was extended to 16.75 m in width after expansion, and the distance between the two centerlines was extended to 37.97 m after expansion.

**Figure 3:**
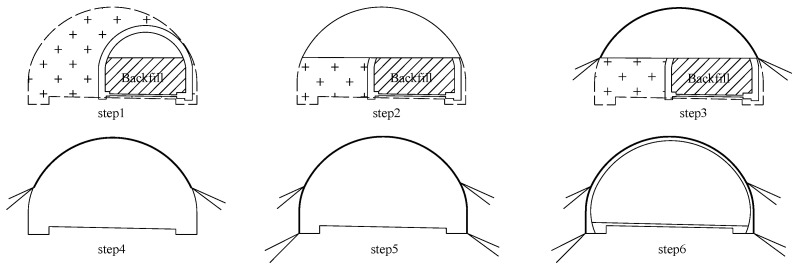
The excavation sequence for the in-situ expansion of the north tunnel. The dashed areas represents the lining profile after in-situ expansion and the “+” areas represent the unexcavated rock mass.

The surrounding ground mainly consisted of tuff lava (ranging from slightly weathered to strongly weathered) and was classified into grade II or grade III, according to relative specifications. The top-bench excavation method was adopted and the supporting system was designed according to the principle of the New Austrian Tunnelling Method (NATM). The shotcrete and foot-lock bolt were used as a primary lining, and the reinforced concrete was cast in place as a secondary lining. Taking mileage NK18+100~300 of the north tunnel as an example, the excavation sequence was schematically shown in Figure 3.

Step 1: The bench part of the existing tunnel was backfilled, which provided a work platform for the top part excavation.

Step 2: The rock mass, as well as the existing tunnel lining of the top part, was excavated.

Step 3: The primary lining of the top part was installed.

Step 4: The rock mass, the backfill, as well as the existing tunnel lining of the bench part, were excavated.

Step 5: The primary lining of the bench part was installed.

Step 6: The waterproof layer was installed and the reinforced concrete was cast in place as a secondary lining.

Controlling the vibration effect on the adjacent environment was the key issue in urban areas. According to design documents and municipal regulations, the maximum velocity of the adjacent tunnel (alternately accommodating two-direction transportation during construction) was restricted to 5 cm/s. Therefore, the traditional blast scheme, as well as the non-cut blast scheme, were both proposed in this article and described in detail as follows.

### 2.2. Traditional Blast Scheme

**Figure 4:**
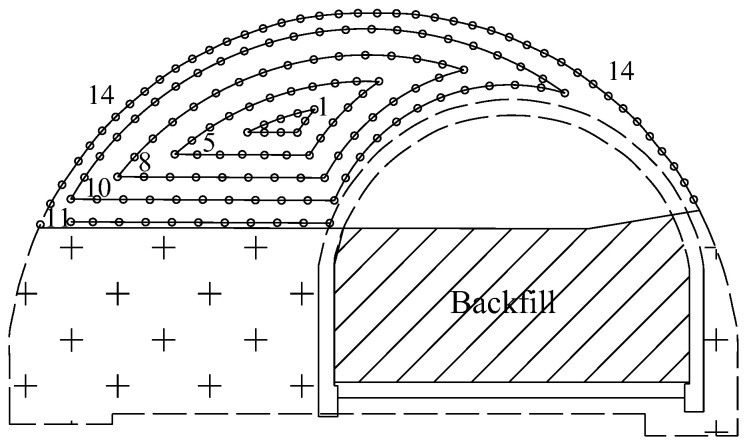
Traditional blast scheme for the top part of the north tunnel. Numbers represent detonator sequences. Plus sign represents unexcavated rock mass. Circles represent blast holes.

Taking mileage NK18+110 of the north tunnel as an example, the traditional blast scheme for the top part is illustrated in Figure 4, and the detailed parameters for blast holes are listed in Table 1. Each blast hole has the same diameter of 42 mm, and the designed cycle footage was 2.0 m. The depths of the auxiliary hole and peripheral hole were 2.1 m, and the depths of the “V”-type cutting hole was 2.3 m. A 2# emulsion explosive with diameter of 32 mm was used, which was initiated from the bottom by an electronic detonator. The explosive consumption for one standard cycle was 134.8 kg, and the explosive consumption per volume of rock mass was 1.35 kg/m^3^.

### 2.3. Non-cut Blast Scheme

**Figure 5:**
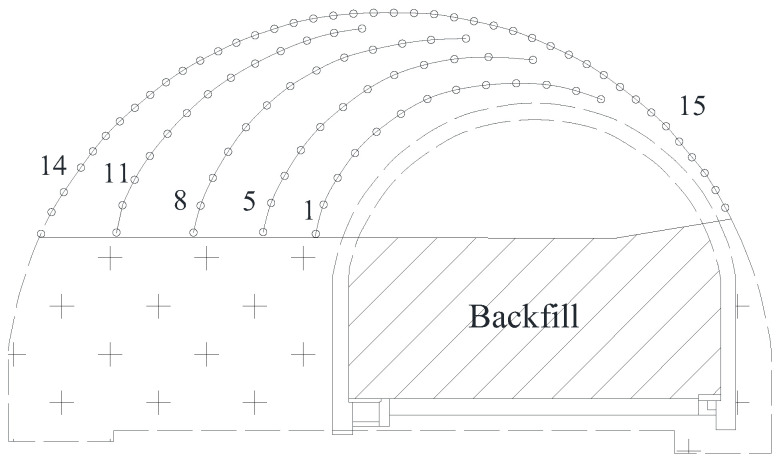
Non-cut blast scheme for the top part of the north tunnel.

Considering the free surface of the existing tunnel, an optimized blast scheme without cutting hole was also proposed in this article, as illustrated in Figure 5. Instead of creating a free surface by cutting holes, the non-cut blast scheme took advantage of the existing free surface, and the arrangement of blast holes was basically parallel to the free surface (i.e., existing tunnel). The designed cycle footage and the depth of the blast hole were the same as in a traditional blast scheme, and the detailed parameters for blast holes were listed in Table 2.

The maximum charge for one single sequence was reduced by 57.6%, the explosive consumption for one standard cycle was reduced by 50.3% (to 67.0 kg), and the explosive consumption per unit rock mass was reduced to 0.67 kg/m^3^.

## 3. Numerical Simulation for the Millisecond Blasting of Tunnel Excavation

### 3.1. Dynamic Calculation in FLAC^3D^

FLAC^3D^ is a fast Lagrange analysis program based on a 3D finite difference method developed by ITASCA. The program discretizes the solution domain based on a difference grid, and represents the differential equations required to be solved using finite and discrete difference equations. In the discretization process, the program uses the difference quotient approximation instead of the inverse in the differential equations to obtain the difference decomposition used to approximate the solution of the differential equations. The solution process of FLAC^3D^ adopts explicit methods, which effectively reduce memory and computation time consumption when solving large-scale problems.

The dynamic analysis module of FLAC^3D^ is also based on the explicit difference algorithm, which realizes the solution of all equations of motion by concentrating the surrounding mesh density over the mesh point mass. This method is capable of coupling rock-soil mass and structural units, making it highly suitable for analyzing soil structure-interaction issues induced by events such as earthquakes, blasting, and so on.

**Figure 6:**
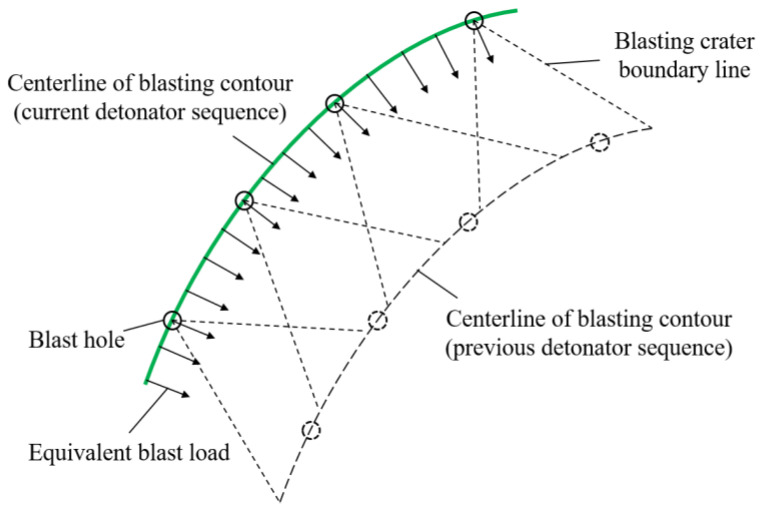
Loading boundary of equivalent blasting load.

When performing a blast vibration analysis in FLAC, the equivalent blast load could be applied to the centerline of blasting contour (i.e., the expected excavation contour), as shown in Figure 6. The equivalent blast load was loaded as follows:

Step 1: According to the position of the blast hole and the range of the rock mass to be blasted, the loading interface of explosion load was determined, and the palm face was divided into several pre-blasting areas.

Step 2: After an explosive blast in a section, the rock mass was considered to be perfectly flaked off from the area controlled by the blast hole in that section, and the grids of this area were deleted by the “Delete” command.

Step 3: The required equivalent blast loads were inputted using the “Table” command and applied to each of the reserved blast load loading interfaces using the “Apply” command.

### 3.2. Numerical Model

**Figure 7:**
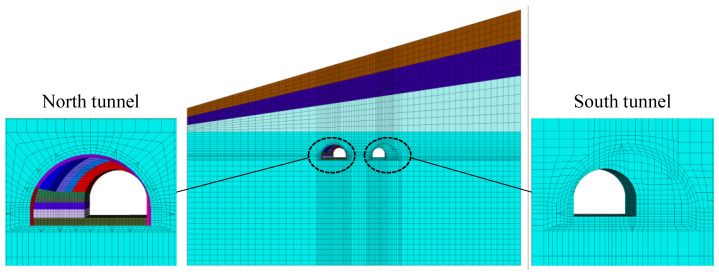
Numerical model for the Gushan tunnel.

The numerical model for the Gushan tunnel was established in the FLAC^3D^ platform. The overall size of the numerical model was 250 m × 4 m × 117 m/190 m (width × depth × height left/right), as shown in Figure 7. The strata consisted of fully, strongly, moderately, and slightly weathered tuff lava, where the ground surface was slightly biased with an angle of 16°. The tunnel had an average overburden of 65 m and was mainly located in the stratum of slightly weathered tuff lava.

The rock mass was simulated by hexahedral grids, with a total of 96,698 zones and 105,015 nodes. The Mohr-Coulomb constitutive model was adopted for each stratum, with the mechanical properties listed in Table 3. The existing tunnel lining was simulated by 3332 liner elements, with the mechanical properties also listed in Table 3.

The ground is typically regarded as an infinite medium when solving geotechnical engineering problems. The border far from the area of interest is generally configured as a fixed and elastic boundary in numerical simulation. In dynamic problems, however, the fixed and elastic boundary may cause the “unreal” reflection of vibration wave. The FLAC^3D^ platform provides a quiet boundary to absorb vibration energy and prevent “unreal” reflection, which can reasonably simulate the infinite rock mass. 

The essence of a quiet boundary is to set a normal damper and a tangential damper on the boundary so that the vibration wave can be absorbed or canceled. The normal and tangential viscous forces (i.e., $\tau_{n}$ and $\tau_{s}$) provided by the dampers could be calculated by [38].

(1) $$\left\{ \begin{array}{l} \tau_{n}=-\rho C_{p}v_{n}\\ \tau_{s}=-\rho C_{s}v_{s} \end{array}\right.$$

where, *v_n_* and *v_s_* were the normal and tangential velocities on the model boundary, respectively, *ρ* was the density of rock mass, and *C_p_* and *C_s_* were the compression and shear wave velocities of the stratum, respectively.

### 3.3. Equivalent Blasting Load for Two Blast Schemes

The instantaneous blasting process can be generally simplified as a triangular impulse load. For the ground classification of grade II and the 2# emulsion explosive, Guan et al. [39] provided an empirical formula to estimate the peak and the duration of the equivalent blasting load. The empirical formula, as shown in Equation (2), was fitted from the refined numerical simulations. The fluid–solid interaction algorithm performed on LS DYNA was used to simulate the transient process of blasting. The pressure–time history curve on the crushing zone boundary of blast hole was acquired, and the simplified triangular impulse load could be fitted for far-field vibration effect evaluation.

(2) $$\left\{ \begin{array}{l} P_{\max}=187K_{l}^{-0.996}\\ t=305K_{l}^{-0.587} \end{array}\right.$$

where, *P*_max_ (in MPa) and *t*_dur_ (in μs) were the peak pressure and the duration of equivalent blasting load, *K_l_* (dimensionless) was the axial decoupling coefficient of the blast hole and indicated the charging quantity compared to blast hole length.

**Figure 8:**
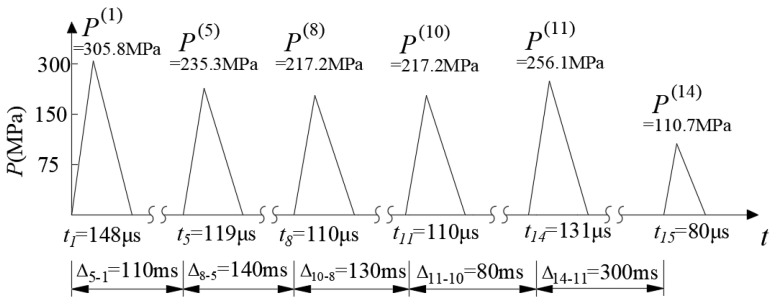
Equivalent blasting loads for every detonator sequence in a traditional blast scheme.

According to Equation (2) and Table 1, the equivalent blasting loads for every detonator sequence in a traditional blast scheme could be calculated and shown in Figure 8. It was notable that the time interval between adjacent detonator sequences (in milliseconds) was much larger than the duration of the equivalent blasting load (in microseconds). Therefore, the breaking in the horizontal axis (time axis) indicated the dull time without impulse load. Taking denotator sequence 8 as an example, the trigger time was 250 ms after detonation, and the peak and duration of the equivalent blasting load were 217.2 MPa and 110 μs, respectively.

**Figure 9:**
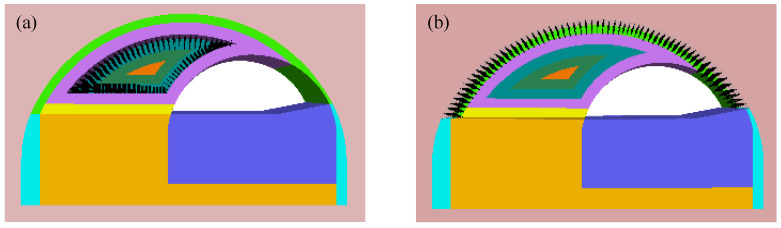
The implementation of equivalent blasting load for traditional blast scheme: (**a**) detonator sequence 8; (**b**) denotator sequence 14.

According to the excavation sequence shown in Figure 3, the bench part of the existing tunnel was backfilled first, and the top part of the existing tunnel lining was then removed from the numerical model. Subsequently, the equivalent blasting load of each detonator sequence (as shown in Figure 8) was applied to the centerline of the blasting contour (i.e., the centerline of the blast holes) in the numerical model according to the loading method in Section 3.1, chronologically. Taking detonator sequence 8 and sequence 14 as examples, the implementation of equivalent blasting loads in the traditional blast scheme was shown in Figure 9. The specific steps for implementing the equivalent blast load were as follows:

Step 1: Backfilled the bench part of the existing tunnel and then removed the existing lining of the top part.

Step 2: Cutting hole blasting (detonator sequence 1), removing the rock mass within the blasting contour line of the cutting hole, and loading the equivalent cutting hole blasting load on the centerline of the blasting contour.

Step 3: Auxiliary hole blasting (detonator sequences 5, 8, 10, and 11), deleting the rock mass within the blasting contour line of each auxiliary hole, and loading the equivalent blasting load of the corresponding segment on the centerline of the blasting contour.

Step 4: Peripheral hole blasting (detonator sequence 14), deleting the rock mass within the blasting contour line of the peripheral hole, and loading the peripheral hole equivalent blasting load on the centerline of the blasting contour.

**Figure 10:**
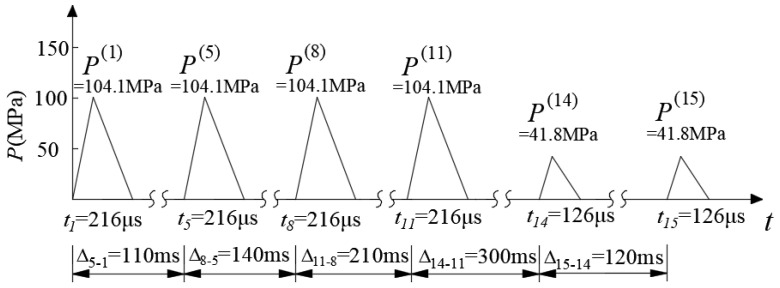
Equivalent blasting load for every detonator sequence in the non-cut blast scheme.

The numerical model and the implementation of equivalent blasting loads for the non-cut blast scheme was basically the same as the traditional blast scheme. According to Equation (2) and Table 2, the equivalent blasting loads for every detonator sequence in the non-cut blast scheme were calculated and shown in Figure 10. For denotator sequence 8, the trigger time was 250 ms after detonation, the peak and duration of the equivalent blasting load were 104.1 MPa and 216 μs, respectively.

**Figure 11:**
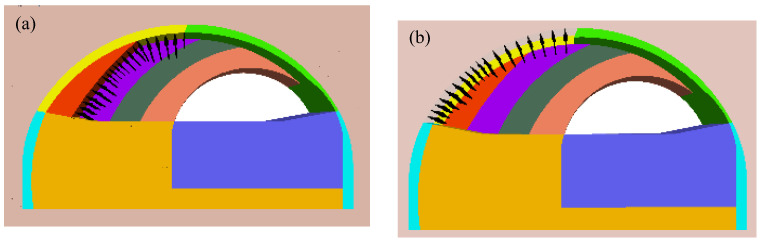
The implementation of equivalent blasting load for the non-cut blast scheme: (**a**) detonator sequence 8; (**b**) detonator sequence 14.

Similarly, the equivalent blasting loads shown in Figure 10 were applied chronologically upon the blasting contour (i.e., the centerline of blast holes) in the numerical model. As an example, the implementation of detonator sequence 8 and sequence 14 was shown in Figure 11. It could be seen from Figure 11 that, due to the change in the arrangement of the blast hole, the pre-division method of rock mass was also different, and the pre-excavation sections of each section were roughly distributed in strips along the outline of the existing tunnel.

### 3.4. Arrangement of Numerical Monitoring Points

**Figure 12:**
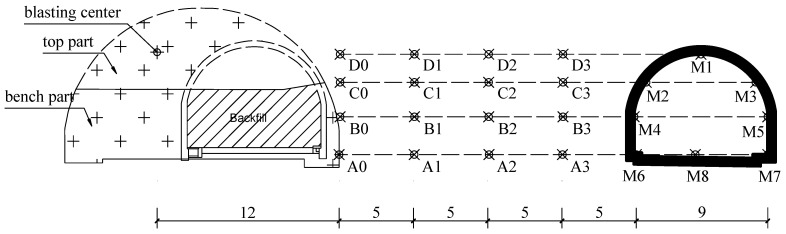
Arrangement of numerical monitoring points (unit: m).

Monitoring points were set at key nodes in the numerical model by using the “model history” command to record the vibration response of rock mass and existing structures during the expansion blasting process. As shown in Figure 12, eight monitoring points denoted by M1~M8 were deployed on the adjacent tunnel, and sixteen monitoring points denoted by A0~A3, B0~B3, C0~C3, and D0~D3 were deployed within the interlaid rock mass. The velocity–time histories of every monitoring point during the whole blasting process were recorded, and the vibration effect on the adjacent tunnel and the vibration effect within the interlaid rock mass are carefully studied in the next section.

## 4. Blasting Vibration Analyses Based on Numerical Simulations

### 4.1. Vibration Effect on Adjacent Tunnel

**Figure 13:**
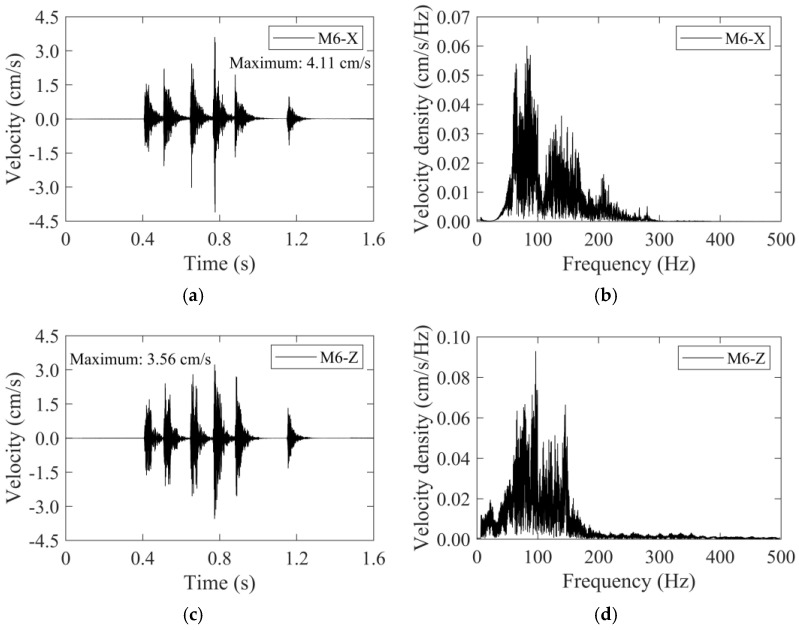
The velocity–time histories and frequency spectra of the M6 monitoring point for the traditional blast scheme: (**a**) time history in the *X*-direction, (**b**) frequency spectrum in the *X*-direction, (**c**) time history in the *Z*-direction, (**d**) frequency spectrum in the *Z*-direction.

For the traditional blast scheme, the velocity–time histories of the M6 monitoring point were depicted in Figure 13 as examples. The time histories were characterized by multi-peaks, which roughly corresponded to the six detonator sequences in the traditional blast scheme. The maximum velocities occurred at detonator sequence 10#, with their values also being listed in Figure 13. The vibration effects in the *X*-direction and *Z*-direction were strongly correlated and kept changing synchronously. Fast Fourier Transform was used to obtain the frequency spectrum of the simulated vibration velocity, as shown in Figure 13b,d. The spectrum analysis showed that the velocity density was concentrated on the frequency range of 50 Hz~170 Hz for the *X*-direction, and the frequency range of 50 Hz~150 Hz for the *Z*-direction.

**Figure 14:**
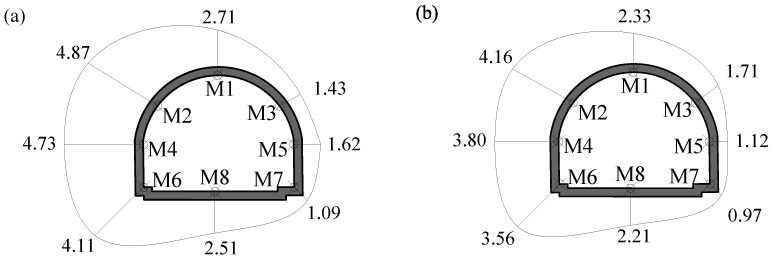
The maximum velocities on the adjacent tunnel for the traditional blast scheme: (**a**) the *X*-direction; (**b**) the *Z*-direction. Units: cm/s.

Furthermore, the maximum velocities on the adjacent tunnel (i.e., M1~M8 monitoring points) were depicted in Figure 14. The maximum velocities on the left side (i.e., blast side) were much larger than their counterparts on the right side (i.e., back side). The maximum velocity occurred at point M2 (the left arch shoulder), with its value reaching 4.87 cm/s in the *X*-direction and 4.16 cm/s in the *Z*-direction, respectively, which were both very close to the safety threshold.

**Figure 15:**
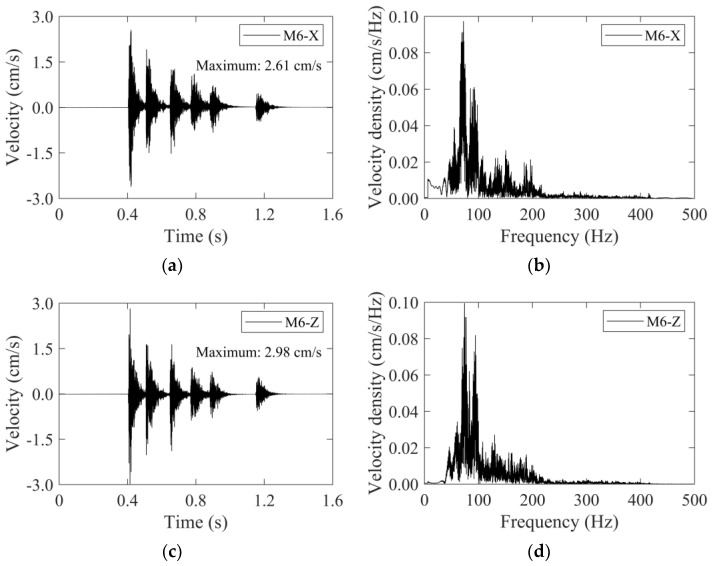
The velocity–time histories and frequency spectra of the M6 monitoring point for the non-cut blast scheme: (**a**) time history in the *X*-direction, (**b**) frequency spectrum in the *X*-direction, (**c**) time history in the *Z*-direction, (**d**) frequency spectrum in the *Z*-direction.

For the non-cut blast scheme, the velocity–time histories of the M6 monitoring point were also depicted in Figure 15. The time histories were also characterized by multi-peaks, and roughly corresponded to the six detonator sequences in the non-cut blast scheme. Due to the small charge quantity in peripheral holes and the short time interval between detonator sequences, the peaks of detonator sequences 14# and 15# were difficult to distinguish. The maximum velocities occurred at detonator sequence 1#, with their values also being listed in Figure 15. Similarly, the frequency spectrum analysis was conducted as shown in Figure 15b,d. The frequency spectrum had an identical concentrated range for velocity in the X-direction and the Z-direction, which was 50 Hz~120 Hz. The frequency spectrum of the traditional cut scheme was close to that of the non-cut scheme. 

**Figure 16:**
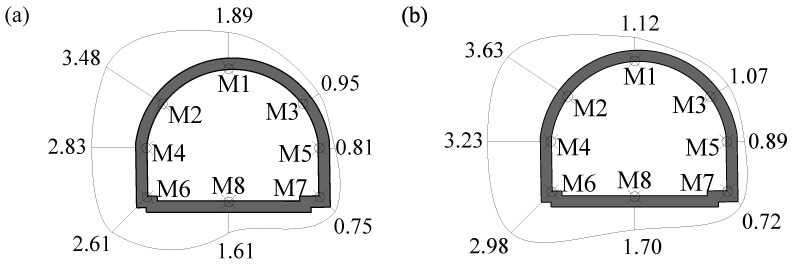
The maximum velocities on the adjacent tunnel for the non-cut blast scheme: (**a**) the *X*-direction; (**b**) the Z-direction; units: cm/s.

The maximum velocities on adjacent tunnel were also depicted in Figure 16, and the distribution of maximum velocities along the adjacent tunnel was similar to that in Figure 14. The maximum velocity also occurred at point M2, with its value reaching 3.48 cm/s in the *X*-direction and 3.63 cm/s in the *Z*-direction, respectively, with a large safety margin from the vibration velocity threshold. Compared to the traditional blast scheme, the vibration effect on the adjacent tunnel was roughly reduced by 30~40% by using the non-cut blast scheme, due to the face–parallel arrangement of blast holes and the lower consumption of explosives.

### 4.2. Vibration Effects within Interlaid Rock Mass

**Figure 17:**
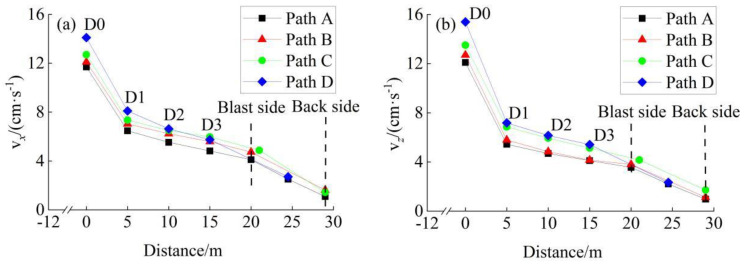
The maximum velocities within interlaid rock mass for the traditional blast scheme: (**a**) *X*-direction, (**b**) Z-direction.

**Figure 18:**
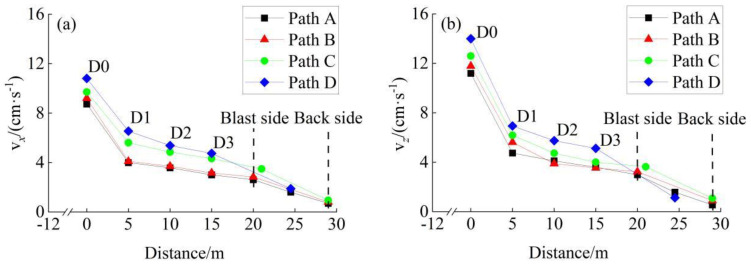
The maximum velocities within the interlaid rock mass for the non-cut blast scheme: (**a**) *X*-direction, (**b**) Z-direction.

The maximum velocities of two blast schemes along four horizontal propagation paths were illustrated in Figure 17 and Figure 18, respectively. Taking the traditional blast scheme as an example, the rock mass near to the blasting area vibrated significantly. The maximum velocity at the D0 measuring point reached 14.88 cm/s in the *X*-direction and 15.25 cm/s in the *Z*-direction. However, the maximum velocity declined rapidly within the propagation path of 0–5 m, where the average attenuation rate reached 1.083 cm/s (in the *X*-direction) and 1.422 cm/s (in the *Z*-direction) per meter. The attenuation rate decreased significantly within the propagation path of 5–20 m, and then increased slightly within the propagation path of 20–29 m (passing through the existing tunnel cavities). The case of the non-cut blast scheme was similar to that presented above, but not mentioned in detail. In a word, whether it was the traditional blast scheme or the non-cut blast scheme proposed in this paper, the attenuation law of vibration velocity of interlaid rock mass in the *X*-direction and the *Z*-direction showed a logarithmic form of rapid attenuation followed by slow attenuation, which also showed that the existing lining and cavity had a strong weakening effect on the vibration.

Generally speaking, if the maximum velocity exhibited a logarithmic attenuation trend along the propagation path, then it could be fitted by Equation (3).

(3) v=αln(d+β)+γ

where *v* denotes the maximum velocity, *d* denotes the horizontal distance of propagation, and *α*, *β*, and *γ* are fitting parameters. The fitting result of maximum velocity along path A (as shown in Figure 17 and Figure 18) is shown in Table 4, where the Root Mean Square Error (RMSE) and Determination Coefficient (R^2^) are used as the fitting evaluation indicators. None of the RMSEs of the R^2^ along path A exceeded 0.61 cm/s or 0.96, respectively. The fitting results for paths B, C, and D were similar to those of path A, which are not listed in Table 4.

### 4.3. Comparison and Summary of Two Blast Schemes

The main parameters and results of the traditional blast scheme and the non-cut blast scheme are summarized in Table 5. Among them, the non-cut blast scheme made full use of the existing tunnel-free surface and relied on the gravity of rock mass to achieve falling, which significantly reduced the amount of explosives needed. The total amount of explosives was reduced by about 50%, and the maximum amount of explosives in a single sequence was reduced by about 60%.

Based on the vibration velocity of the adjacent tunnel, the vibration intensity distribution of the two blast schemes on the lining was basically consistent, and the maximum vibration velocity was obtained at the arch shoulder of the blast side. Among them, the vibration of the non-cut blast scheme was reduced by approximately 28.54% in the *X*-direction and 12.74% in the *Z*-direction at the blast side’s arch shoulder, and the average vibration reduction amplitudes of each point of the adjacent tunnel lining in the *X*- and *Z*-directions were approximately 35.76% and 25.35%, respectively. According to the average attenuation rate of interlaid rock mass in Table 5, it could be considered that the vibration attenuation velocity of the two blast schemes in the *X*-direction and *Z*-direction was basically consistent.

In general, the non-cut blast scheme was a simple blast hole arrangement, and could make full use of the existing free surface to enhance the rock-breaking effect and achieve the rock falling by its own gravity, which greatly saved the amount of explosives and made the blasting vibration lighter, so it was obviously more suitable for in situ expansion projects.

## 5. Blasting Vibration Analyses Based on Field Monitoring

**Figure 19:**
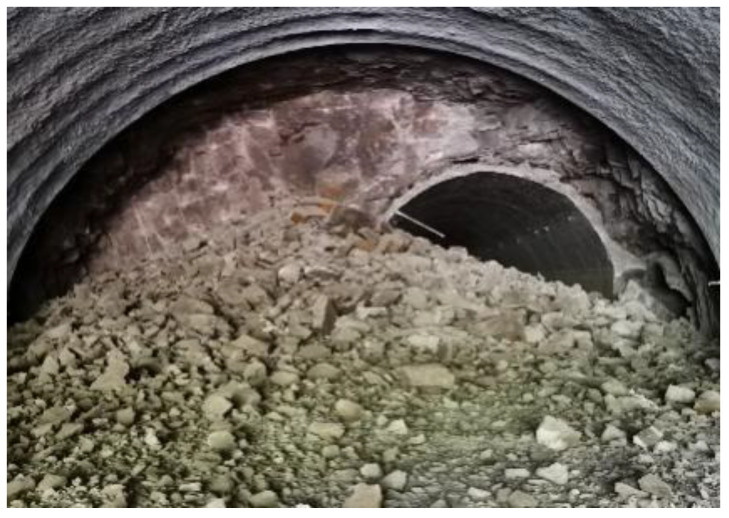
The upper part of NK18+110 section before and after expansion.

According to the numerical simulations as presented above, the non-cut blast scheme was superior to the traditional blast scheme in terms of reducing explosive consumption and vibration effect. Therefore, the non-cut blast scheme was put into practice for the in-situ expansion project of the Gushan Tunnel, and the upper part of the NK18+110 section before and after expansion is illustrated in Figure 19.

**Figure 20:**
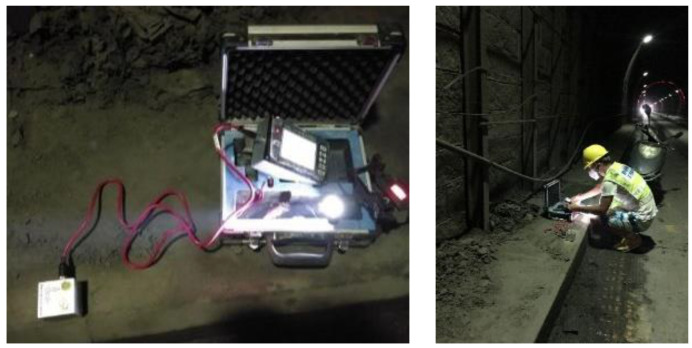
Field monitoring for blasting vibration.

**Figure 21:**
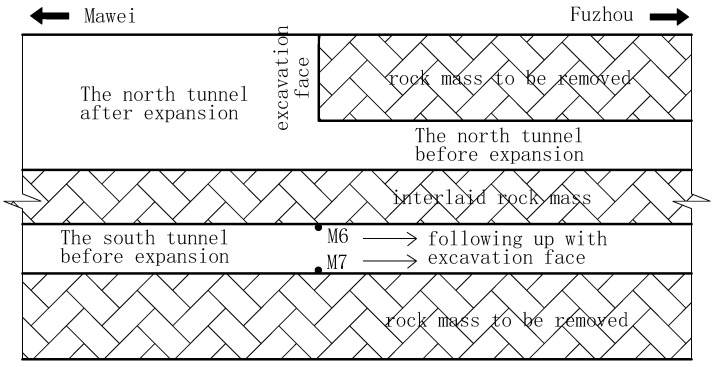
Arrangement of blasting vibration meters.

To assess the vibration effect on the adjacent tunnel and validate the efficiency of the non-cut blast scheme, filed monitoring was also conducted during the in situ expansion of the north tunnel. Two blasting vibration meters were installed at the feet of the adjacent tunnel, as shown in Figure 20. The vibration meter was composed of a micro-computer and two sensors. Among the two sensors, one was the vibration sensor monitoring the velocity, and the other was the sound sensor monitoring the noise in the environment. The computer surrogated the monitoring of the two sensors and transferred the monitoring data to the remote platform wirelessly. The velocity sensing range was 0.0005~35 cm/s, and the frequency sensing range was 5~500 Hz. The two vibration meters corresponded to the M6 point (on the blast side) and M7 point (on the back side) in numerical simulations, respectively. The vibration meters followed up with the advancement of the north tunnel excavation face, as seen in Figure 21. A total of 136 cycles were carried out in the north tunnel excavation (from NK18+100 to NK+300 with ground classification of grade II). As far as 42 standard footage cycles in the upper part of north tunnel were concerned, 84 monitoring data (velocity–time histories) were recorded.

**Figure 22:**
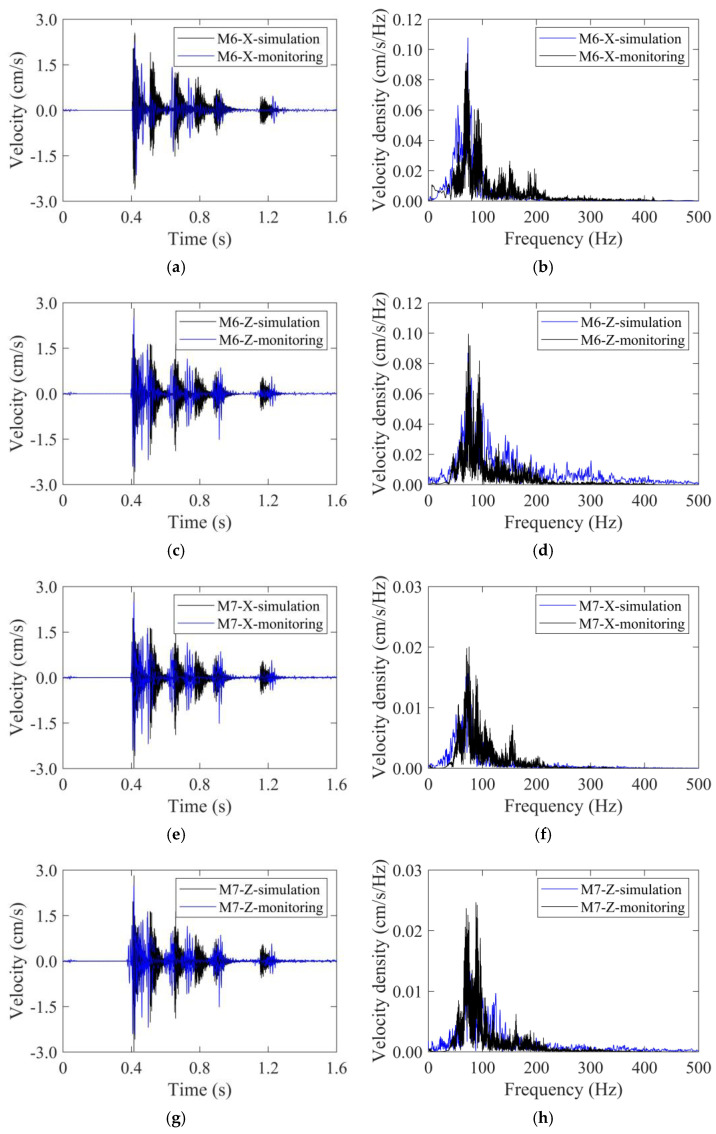
The velocity–time histories and frequency spectra recorded by field monitoring and compared with numerical simulation results: (**a**) time history of M6 in the *X*-direction, (**b**) frequency spectrum of M6 in the *X*-direction, (**c**) time history of M6 in the Z-direction, (**d**) frequency spectrum of M6 in the Z-direction, (**e**) time history of M7 in the *X*-direction, (**f**) frequency spectrum of M7 in the *X*-direction, (**g**) time history of M7 in the Z-direction, (**h**) frequency spectrum of M7 in the Z-direction.

Taking the upper part of the NK18+110 section as an example, the velocity–time histories obtained from field monitoring are depicted in Figure 22. Similar to the simulation results, the time histories were also characterized by multi-peaks, and generally corresponded to five detonator sequences. The maximum velocities at the left foot (blast side) were 2.23 cm/s in the *xX*-direction and 2.52 cm/s in the *Z*-direction, and occurred at detonator sequence 1#. The maximum velocities at the right foot (back side) were 0.85 cm/s in the *X*-direction and 0.82 cm/s in the *Z*-direction, much smaller than their counterparts at the left foot.

The velocity–time histories corresponding to 42 standard footage cycles were all recorded in a similar way, with their maximum velocities being summarized in Table 6. The monitoring data showed that the maximum velocities of the south tunnel were all less than 3 cm/s and under the safety threshold of 5 cm/s, which implied that the non-cut blast scheme was more suitable for the in situ tunnel expansion projects.

Furthermore, the maximum velocities recorded from 42 standard footage cycles were averaged to 2.44 cm/s (M6 in the *X*-direction), 2.51 cm/s (M6 in the *Z*-direction), 0.76 cm/s (M7 in the *X*-direction), and 0.77 cm/s (M7 in the *Z*-direction), respectively. The maximum velocities on the blast side being slightly overestimated by 7.0%. The simulated time histories of vibration velocity agreed well with the field monitoring results from Figure 22a,c,e,g. Furthermore, the simulated frequency spectrum also agreed with the field monitoring results. The concentrated frequency ranges for both monitored and simulated velocity were 50 Hz~120 Hz. The velocity spectrum density of point M6 was much higher than that of point M7. This observation was in accordance with time history analysis.

## 6. Conclusions

Based on the in situ expansion project of the Gushan Tunnel in Fuzhou, the traditional and optimized blast schemes were thoroughly compared through refined numerical simulations. The vibration attenuation within the interlaid rock mass and the vibration effect on the adjacent tunnel lining were investigated. The optimized non-cut blast scheme was then put into practice, and the vibration effect on the adjacent tunnel lining was monitored. Some conclusions could be drawn as follows:

(1) Applying equivalent blasting loads upon the blasting contour by detonator sequences could simulate the whole process of millisecond blasting, which could be used as an effective method for blast scheme optimization and vibration effect prediction.
(2) Both the simulation and the monitoring results showed that the vibration effect on the adjacent tunnel’s back side was much smaller than its counterpart on the blast side. In particular, the presence of a cavity considerably reduced the blasting vibration effect.
(3) The non-cut blast scheme, which effectively utilized the existing free surface, could reduce the explosive consumption and vibration effect significantly, and might be preferred for the tunnel in situ expansion projects.

In a further study, the detailed layout of blast holes could be optimized through a series of numerical simulations, and the proposed non-cut construction method might be validated in more field cases.

## Figures and Tables

**Table 1:** Blast hole parameters for the traditional blast scheme.

	Detonator Sequence	Hole Distance	Number of Hole	Delayed Time	Charge Quantity per Hole	Axial Decoupling Coefficient *K_l_*	Charge Quantity per Sequence
Cutting hole	1	0.5 m	9	0 ms	1.7 kg	1.3	15.5 kg
Auxiliary hole	5	0.6 m	19	110 ms	1.2 kg	1.8	23.2 kg
	8	0.6 m	28	250 ms	1.1 kg	2.0	30.8 kg
	10	0.6 m	30	380 ms	1.1 kg	2.0	33 kg
	11	0.6 m	11	460 ms	1.4 kg	1.6	15.1 kg
Peripheral hole	14	0.5 m	43	760 ms	0.4 kg	5.5	17.2 kg

**Table 2:** Blast hole parameters for non-cut blast scheme.

	Detonator Sequence	Hole Distance	Number of Hole	Delayed Time	Charge Quantity per Hole	Axial Decoupling Coefficient *K_l_*	Charge Quantity per Sequence
Auxiliary hole	1	0.6 m	12	0 ms	1.0 kg	2.2	12.0 kg
	5	0.6 m	12	110 ms	1.0 kg	2.2	12.0 kg
	8	0.7 m	13	250 ms	1.0 kg	2.2	13.0 kg
	11	0.7 m	14	460 ms	1.0 kg	2.2	14.0 kg
Peripheral hole	14	0.5 m	20	760 ms	0.4 kg	5.5	8.0 kg
	15	0.5 m	20	880 ms	0.4 kg	5.5	8.0 kg

**Table 3:** The mechanical properties of rock mass and existing tunnel lining.

	Density *ρ* (g·cm^−3^)	Young’s Modulus *E* (GPa)	Poisson Ratio *μ*	Cohesive Forces *c* (kPa)	Friction Angle *φ* (°)	Thickness (m)
Strongly weathered	2.2	20.5	0.33	690	39.33	
Moderately weathered	2.5	24	0.31	1620	51.45	
Slightly weathered	2.6	26	0.3	1740	51.76	
Existing tunnel lining	2.7	32.2	0.2			0.35

**Table 4:** Fitting results for the peak vibration velocity attenuation on the propagation path.

	Propagation Path	Fitting Equations	RMSE (cm·s^−1^)	*R* ^2^
Figure 17a	path A	$v_{x}=-3.521ln(d+2.041)+14.112$	0.606	0.96
Figure 17b	path A	$v_{z}=-2.473ln(d+0.478)+10.251$	0.553	0.97
Figure 18a	path A	$v_{x}=-1.876ln(d+0.590)+7.719$	0.416	0.97
Figure 18b	path A	$v_{z}=-2.373ln(d+0.476)+9.405$	0.552	0.97

**Table 5:** Main parameters and results of the two blast schemes.

	Traditional Scheme	Optimized Scheme	Remark
Total charge (kg)	134.8	67.0	50.2% reduction
Maximum charge in single sequence (kg)	33.0	14.0	57.6% reduction
Maximum vibration velocity of monitoring points of adjacent tunnel lining (*X*-direction) (cm/s)	2.71 (M1) 4.87 (M2) 1.43 (M3) 4.73 (M4) 1.62 (M5) 4.11 (M6) 1.09 (M7) 2.51 (M8)	1.89 (M1) 3.48 (M2) 0.95 (M3) 2.83 (M4) 0.81 (M5) 2.61 (M6) 0.75 (M7) 1.61 (M8)	35.76% reduction in average
Maximum vibration velocity of monitoring points of adjacent tunnel lining (*Z*-direction) (cm/s)	2.33 (M1) 4.16 (M2) 1.71 (M3) 3.80 (M4) 1.12 (M5) 3.56 (M6) 0.97 (M7) 2.21 (M8)	1.12 (M1) 3.63 (M2) 1.07 (M3) 3.23 (M4) 0.89 (M5) 2.98 (M6) 0.72 (M7) 1.70 (M8)	25.35% reduction in average
Average attenuation rate of interlaid rock mass (*X*-direction) (cm·s−1/m)	(0–5 m): 1.083 (5–20 m): 0.159 (20–29 m): 0.354	(0–5 m): 0.911 (5–20 m): 0.106 (20–29 m): 0.239	Basically consistent
Average attenuation rate of interlaid rock mass (*Z*-direction) (cm·s^−1^/m)	(0–5 m): 1.422 (5–20 m): 0.146 (20–29 m): 0.286	(0–5 m): 1.305 (5–20 m): 0.149 (20–29 m): 0.271	Basically consistent

**Table 6:** Maximum velocities of M6 and M7 monitoring points.

Monitoring Point	Mileage	In *X*-Direction (cm·s^−1^)	In *Z*-Direction (cm·s^−1^)
M6	NK18+104	2.44	2.61
	NK18+110	2.23	2.52
	NK18+214	2.52	2.98
	…	…	…
	averaged	2.44	2.51
M7	NK18+104	0.73	0.70
	NK18+110	0.85	0.82
	NK18+214	0.75	0.54
	…	…	…
	averaged	0.76	0.77

## Data Availability

The data used to support the findings of this study are included within the article.
